# 3-(4-Methoxy­phen­yl)isochroman-1-one

**DOI:** 10.1107/S1600536808032625

**Published:** 2008-10-15

**Authors:** Muhammad Tahir Hussain, M. Nawaz Tahir, Ahmad Adnan, Asif Hanif Ch, Nasim Hassan Rama

**Affiliations:** a Department of Chemistry, National Textile University, Faisalabad, Pakistan; b Department of Physics, University of Sargodha, Sargodha, Pakistan; c Department of Chemistry, Government College University, Lahore, Pakistan; d Department of Chemistry, Quaid-i-Azam University, Islamabad 45320, Pakistan

## Abstract

In the mol­ecule of the title compound, C_16_H_14_O_3_, the aromatic rings are oriented at a dihedral angle of 72.02 (6)°. The heterocyclic ring adopts a twisted conformation. In the crystal structure, there are C—H⋯π contacts between the heterocyclic and phenyl rings, and between the methyl group and methoxy­phenyl ring.

## Related literature

For related structures, see: Schmalle *et al.* (1982 ▶); Schnebel *et al.* (2003 ▶). For bond-length data, see: Allen *et al.* (1987 ▶). For ring puckering parameters, see: Cremer & Pople (1975 ▶). For a description of the Cambridge Structural Database, see: Allen (2002 ▶).
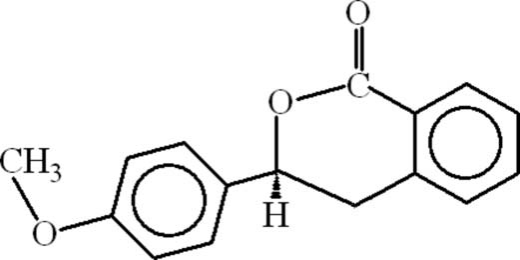


## Experimental

### 

#### Crystal data

C_16_H_14_O_3_
*M* *_r_* = 254.27
Monoclinic, 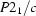

*a* = 11.8933 (7) Å
*b* = 14.6874 (9) Å
*c* = 7.4521 (4) Å
β = 101.040 (2)°
*V* = 1277.66 (13) Å^3^
*Z* = 4
Mo *K*α radiation
μ = 0.09 mm^−1^
*T* = 296 (2) K
0.24 × 0.16 × 0.12 mm

#### Data collection

Bruker KappaAPEXII CCD diffractometer
Absorption correction: multi-scan (*SADABS*; Bruker, 2005 ▶) *T*_min_ = 0.980, *T*_max_ = 0.996
12426 measured reflections
3302 independent reflections
2275 reflections with *I* > 2σ(*I*)
*R*_int_ = 0.023

#### Refinement

*R*[*F* ^2^ > 2σ(*F* ^2^)] = 0.042
*wR*(*F* ^2^) = 0.117
*S* = 1.01
3302 reflections
175 parameters
H atoms treated by a mixture of independent and constrained refinement
Δρ_max_ = 0.14 e Å^−3^
Δρ_min_ = −0.22 e Å^−3^

### 

Data collection: *APEX2* (Bruker, 2007 ▶); cell refinement: *APEX2*; data reduction: *SAINT* (Bruker, 2007 ▶); program(s) used to solve structure: *SHELXS97* (Sheldrick, 2008 ▶); program(s) used to refine structure: *SHELXL97* (Sheldrick, 2008 ▶); molecular graphics: *ORTEP-3 for Windows* (Farrugia, 1997 ▶) and *PLATON* (Spek, 2003 ▶); software used to prepare material for publication: *WinGX* (Farrugia, 1999 ▶) and *PLATON*.

## Supplementary Material

Crystal structure: contains datablocks global, I. DOI: 10.1107/S1600536808032625/hk2543sup1.cif

Structure factors: contains datablocks I. DOI: 10.1107/S1600536808032625/hk2543Isup2.hkl

Additional supplementary materials:  crystallographic information; 3D view; checkCIF report

## Figures and Tables

**Table 1:** Hydrogen-bond geometry (Å, °) *Cg*1 and *Cg*2 are the centroids of the C2–C7 and C10–C15 rings, respectively.

*D*—H⋯*A*	*D*—H	H⋯*A*	*D*⋯*A*	*D*—H⋯*A*
C8—H8*B*⋯*Cg*2^i^	0.97	2.94	3.8398 (16)	154
C16—H16*B*⋯*Cg*3^ii^	0.96	2.89	3.7804 (17)	154

Symmetry codes: (i) 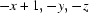; (ii) 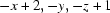.

**Fig. 1.**
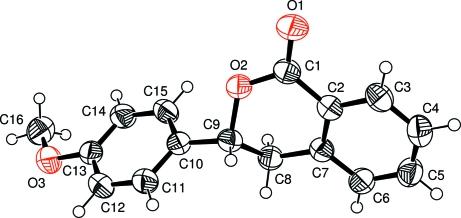
The molecular structure of the title molecule, with the atom-numbering scheme. Displacement ellipsoids are drawn at the 50% probability level.

**Fig. 2.**
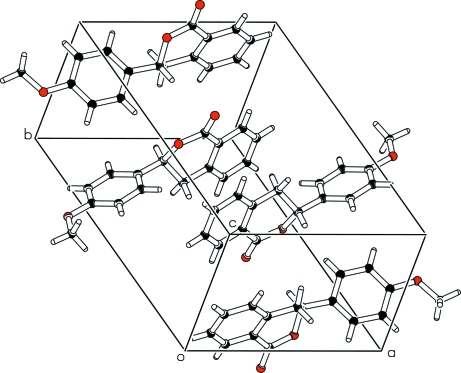
A packing diagram of the title compound.

**Fig. 3.**
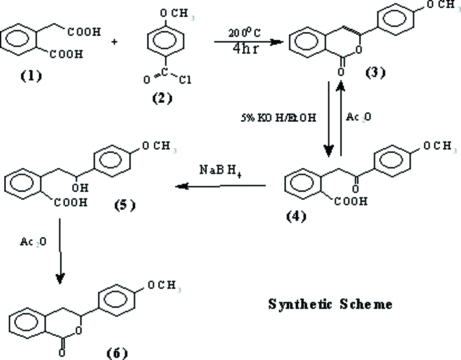
Preparation of the title compound.

## supplementary crystallographic information

### Comment

The title compound was prepared in order to evalute its potential as antibacterial and antifungal agents. The CCDC search (Allen, 2002) showed that the crystal structures of rac-*exo*-tricarbonyl-(h6-3-phenylisochromanone)-chromium (Schnebel *et al.*, 2003) and 3,4-dihydro-8-hydroxy-3-(4-hydroxyphenyl)-isocoumarin (Schmalle *et al.*, 1982) have been reported, which have close resemblance as far as isochromane and attached phenyl ring is considered.

In the molecule of the title compound (Fig. 1), the bond lengths (Allen *et al.*,  1987) and angles are within normal ranges. Rings A (C2–C7) and C (C10–C15) are, of course, planar, and they are oriented at a dihedral angle of 72.02 (6)°. Ring B (O2/C1/C2/C7–C9) is not planar, having total puckering amplitude, Q_T_, of 0.483 (2) Å and twisted conformation [φ = 41.63 (3)° and θ = 116.64 (3)°] (Cremer & Pople, 1975).

In the crystal structure, there are C—H···π contacts (Table 1) between the heterocyclic and phenyl rings and the methyl group and methoxyphenyl ring, in which they may be effective in the stabilization of the structure.

### Experimental

As shown in Fig. 3, a mixture of homophthalic acid, (1), (1.98 g, 11.0 mmol) and 4-methoxybenzoyl chloride, (2), (7.85 g, 46 mmol) was heated under reflux at 473 K. After concentration, the residue was chromatographed on silica gel column using petroleum ether (333–353 K) to give 3-(4-methoxyphenyl)isocoumarin, (3). 2-[2'-Oxoethyl-2'-(4'-methoxyphenyl)]benzoic acid, (4), was obtained by refluxing a solution of (3) (4 g, 15.9 mmol) in ethanol (200 ml) and potassium hydroxide (5%, 200 ml) for 4 h. NaBH_4_ (1.6 g) was added to a solution of (4) (4.81 g, 17.8 mmol) in sodium hydroxide (1%, 180 ml) and the resulting solution was stirred overnight at room temperature. After being acidified with HCl, the whole mixture was extracted with dichloromethane (2 × 15 ml). Usual work-up gave crude racemic hydroxy-acid, (5), which was dissolved in acetic anhydride (5 ml) and heated under reflux for 2 h to get the title compound (6). The crude compound was purified by column chromatography on silica gel with petroleum ether and recrystallized in ethanol.

### Refinement

H9 atom was located in difference syntheses and refined as [C—H = 0.968 (15) Å; U_iso_(H) = 0.0527 A^2^]. The remaining H atoms were positioned geometrically, with C—H = 0.93, 0.97 and 0.96 Å for aromatic, methylene and methyl H, respectively, and constrained to ride on their parent atoms with U_iso_(H) = xU_eq_(C), where x = 1.5 for methyl H and x = 1.2 for all other H atoms.

### Crystal data

**Table:**

C_16_H_14_O_3_	*F*(000) = 536
*M**_r_* = 254.27	*D*_x_ = 1.332 Mg m^−^^3^
Monoclinic, *P*2_1_/*c*	Mo *K*α radiation, λ = 0.71073 Å
Hall symbol: -P 2ybc	Cell parameters from 3302 reflections
*a* = 11.8933 (7) Å	θ = 2.2–28.8°
*b* = 14.6874 (9) Å	µ = 0.09 mm^−^^1^
*c* = 7.4521 (4) Å	*T* = 296 K
β = 101.040 (2)°	Prismatic, yellow
*V* = 1277.66 (13) Å^3^	0.24 × 0.16 × 0.12 mm
*Z* = 4	

### Data collection

**Table:**

Bruker KappaAPEXII CCD diffractometer	3302 independent reflections
Radiation source: fine-focus sealed tube	2275 reflections with *I* > 2σ(*I*)
graphite	*R*_int_ = 0.023
Detector resolution: 7.40 pixels mm^-1^	θ_max_ = 28.8°, θ_min_ = 2.2°
ω scans	*h* = −16→16
Absorption correction: multi-scan (*SADABS*; Bruker, 2005)	*k* = −19→19
*T*_min_ = 0.980, *T*_max_ = 0.996	*l* = −9→10
12426 measured reflections	

### Refinement

**Table:**

Refinement on *F*^2^	Primary atom site location: structure-invariant direct methods
Least-squares matrix: full	Secondary atom site location: difference Fourier map
*R*[*F*^2^ > 2σ(*F*^2^)] = 0.042	Hydrogen site location: inferred from neighbouring sites
*wR*(*F*^2^) = 0.117	H atoms treated by a mixture of independent and constrained refinement
*S* = 1.01	*w* = 1/[σ^2^(*F*_o_^2^) + (0.0481*P*)^2^ + 0.2427*P*] where *P* = (*F*_o_^2^ + 2*F*_c_^2^)/3
3302 reflections	(Δ/σ)_max_ < 0.001
175 parameters	Δρ_max_ = 0.14 e Å^−^^3^
0 restraints	Δρ_min_ = −0.22 e Å^−^^3^

### Special details

**Table:**

Geometry. Bond distances, angles *etc*. have been calculated using the rounded fractional coordinates. All su's are estimated from the variances of the (full) variance-covariance matrix. The cell e.s.d.'s are taken into account in the estimation of distances, angles and torsion angles
Refinement. Refinement of *F*^2^ against ALL reflections. The weighted *R*-factor *wR* and goodness of fit *S* are based on *F*^2^, conventional *R*-factors *R* are based on *F*, with *F* set to zero for negative *F*^2^. The threshold expression of *F*^2^ > σ(*F*^2^) is used only for calculating *R*-factors(gt) *etc*. and is not relevant to the choice of reflections for refinement. *R*-factors based on *F*^2^ are statistically about twice as large as those based on *F*, and *R*- factors based on ALL data will be even larger.

### Fractional atomic coordinates and isotropic or equivalent isotropic displacement parameters (Å^2^)

**Table:**

	*x*	*y*	*z*	*U*_iso_*/*U*_eq_	
O1	0.56862 (10)	0.12610 (9)	−0.37797 (13)	0.0668 (4)	
O2	0.68506 (8)	0.13694 (7)	−0.11396 (12)	0.0495 (3)	
O3	1.15702 (8)	0.09384 (8)	0.43236 (15)	0.0642 (4)	
C1	0.57829 (12)	0.13397 (9)	−0.21507 (18)	0.0460 (4)	
C2	0.48087 (11)	0.13874 (8)	−0.11934 (17)	0.0421 (4)	
C3	0.37156 (12)	0.15327 (9)	−0.2200 (2)	0.0512 (5)	
C4	0.27961 (12)	0.15613 (10)	−0.1325 (2)	0.0575 (5)	
C5	0.29633 (12)	0.14322 (10)	0.0534 (2)	0.0576 (5)	
C6	0.40443 (12)	0.12764 (10)	0.1541 (2)	0.0519 (5)	
C7	0.49834 (11)	0.12588 (8)	0.06918 (17)	0.0422 (4)	
C8	0.61822 (11)	0.11135 (10)	0.17022 (17)	0.0465 (4)	
C9	0.70140 (11)	0.16318 (9)	0.07877 (17)	0.0440 (4)	
C10	0.82407 (11)	0.14481 (9)	0.16272 (17)	0.0438 (4)	
C11	0.88770 (12)	0.20841 (10)	0.27598 (19)	0.0525 (5)	
C12	0.99780 (12)	0.18919 (11)	0.3646 (2)	0.0573 (5)	
C13	1.04728 (11)	0.10621 (10)	0.34024 (18)	0.0484 (4)	
C14	0.98541 (12)	0.04249 (10)	0.2268 (2)	0.0542 (5)	
C15	0.87482 (12)	0.06259 (10)	0.1397 (2)	0.0529 (5)	
C16	1.20764 (14)	0.00719 (12)	0.4207 (2)	0.0664 (6)	
H3	0.36043	0.16105	−0.34599	0.0615*	
H4	0.20631	0.16679	−0.19903	0.0690*	
H5	0.23396	0.14501	0.11186	0.0691*	
H6	0.41438	0.11826	0.27955	0.0623*	
H8A	0.62502	0.13197	0.29549	0.0558*	
H8B	0.63641	0.04693	0.17237	0.0558*	
H9	0.6859 (12)	0.2278 (10)	0.0814 (19)	0.0527*	
H11	0.85577	0.26487	0.29252	0.0630*	
H12	1.03908	0.23245	0.44148	0.0687*	
H14	1.01772	−0.01364	0.20892	0.0651*	
H15	0.83351	0.01922	0.06319	0.0635*	
H16A	1.28412	0.00733	0.49143	0.0995*	
H16B	1.16306	−0.03839	0.46749	0.0995*	
H16C	1.21001	−0.00605	0.29534	0.0995*	

### Atomic displacement parameters (Å^2^)

**Table:**

	*U*^11^	*U*^22^	*U*^33^	*U*^12^	*U*^13^	*U*^23^
O1	0.0692 (7)	0.0953 (8)	0.0350 (5)	0.0026 (6)	0.0079 (5)	−0.0001 (5)
O2	0.0472 (5)	0.0654 (6)	0.0364 (5)	−0.0014 (4)	0.0096 (4)	0.0001 (4)
O3	0.0460 (6)	0.0818 (8)	0.0601 (6)	0.0054 (5)	−0.0018 (5)	−0.0082 (5)
C1	0.0519 (8)	0.0472 (7)	0.0378 (6)	0.0003 (6)	0.0062 (6)	0.0032 (5)
C2	0.0466 (7)	0.0375 (6)	0.0410 (7)	−0.0019 (5)	0.0057 (5)	0.0020 (5)
C3	0.0539 (8)	0.0477 (8)	0.0480 (8)	−0.0011 (6)	−0.0003 (6)	0.0040 (6)
C4	0.0443 (8)	0.0557 (8)	0.0688 (10)	0.0005 (6)	0.0013 (7)	0.0014 (7)
C5	0.0464 (8)	0.0594 (9)	0.0694 (10)	−0.0051 (6)	0.0170 (7)	−0.0047 (7)
C6	0.0513 (8)	0.0585 (8)	0.0477 (8)	−0.0056 (6)	0.0138 (6)	−0.0006 (6)
C7	0.0446 (7)	0.0408 (6)	0.0409 (7)	−0.0030 (5)	0.0072 (5)	0.0008 (5)
C8	0.0465 (7)	0.0574 (8)	0.0351 (6)	0.0001 (6)	0.0064 (5)	0.0041 (5)
C9	0.0465 (7)	0.0474 (7)	0.0375 (6)	−0.0012 (6)	0.0069 (5)	−0.0026 (5)
C10	0.0435 (7)	0.0493 (7)	0.0396 (6)	−0.0033 (5)	0.0106 (5)	−0.0023 (5)
C11	0.0501 (8)	0.0503 (8)	0.0565 (8)	−0.0003 (6)	0.0089 (6)	−0.0116 (6)
C12	0.0514 (8)	0.0615 (9)	0.0561 (9)	−0.0065 (7)	0.0033 (7)	−0.0168 (7)
C13	0.0416 (7)	0.0636 (9)	0.0401 (7)	−0.0007 (6)	0.0079 (5)	−0.0026 (6)
C14	0.0522 (8)	0.0547 (8)	0.0547 (8)	0.0056 (6)	0.0074 (7)	−0.0085 (7)
C15	0.0514 (8)	0.0522 (8)	0.0525 (8)	−0.0039 (6)	0.0033 (6)	−0.0126 (6)
C16	0.0561 (9)	0.0836 (12)	0.0574 (9)	0.0146 (8)	0.0060 (7)	0.0092 (8)

### Geometric parameters (Å, °)

**Table:**

O1—C1	1.2028 (16)	C12—C13	1.380 (2)
O2—C1	1.3475 (17)	C13—C14	1.376 (2)
O2—C9	1.4636 (15)	C14—C15	1.383 (2)
O3—C13	1.3655 (17)	C3—H3	0.9300
O3—C16	1.418 (2)	C4—H4	0.9300
C1—C2	1.4741 (19)	C5—H5	0.9300
C2—C3	1.387 (2)	C6—H6	0.9300
C2—C7	1.3931 (18)	C8—H8A	0.9700
C3—C4	1.377 (2)	C8—H8B	0.9700
C4—C5	1.375 (2)	C9—H9	0.968 (15)
C5—C6	1.378 (2)	C11—H11	0.9300
C6—C7	1.385 (2)	C12—H12	0.9300
C7—C8	1.4947 (19)	C14—H14	0.9300
C8—C9	1.5102 (19)	C15—H15	0.9300
C9—C10	1.4977 (19)	C16—H16A	0.9600
C10—C11	1.3831 (19)	C16—H16B	0.9600
C10—C15	1.375 (2)	C16—H16C	0.9600
C11—C12	1.378 (2)		
			
O1···C16^i^	3.367 (2)	C16···H14	2.5100
O3···C4^ii^	3.4157 (18)	H3···O1	2.5800
O3···C3^ii^	3.3841 (18)	H3···O3^vi^	2.8300
O1···H3	2.5800	H3···C5^iv^	3.0300
O1···H6^iii^	2.8500	H4···O3^vi^	2.9000
O1···H9^iv^	2.608 (15)	H5···O3^x^	2.8200
O1···H8A^iii^	2.6500	H5···C13^x^	3.1000
O1···H16A^i^	2.8600	H6···O1^xi^	2.8500
O2···H11^iv^	2.6900	H6···H8A	2.4900
O2···H15	2.6400	H8A···O1^xi^	2.6500
O2···H16C^i^	2.7800	H8A···H6	2.4900
O3···H3^ii^	2.8300	H8B···C15	2.9000
O3···H4^ii^	2.9000	H8B···C2^viii^	3.0500
O3···H5^v^	2.8200	H8B···C3^viii^	2.9700
C3···C6^iv^	3.395 (2)	H9···C2	2.915 (14)
C3···O3^vi^	3.3841 (18)	H9···H11	2.3700
C3···C5^iv^	3.465 (2)	H9···O1^vii^	2.608 (15)
C4···O3^vi^	3.4157 (18)	H9···C1^vii^	2.967 (15)
C5···C3^vii^	3.465 (2)	H11···H9	2.3700
C6···C3^vii^	3.395 (2)	H11···O2^vii^	2.6900
C16···O1^i^	3.367 (2)	H14···C16	2.5100
C1···H9^iv^	2.967 (15)	H14···H16B	2.3600
C2···H8B^viii^	3.0500	H14···H16C	2.2600
C2···H9	2.915 (14)	H15···O2	2.6400
C3···H16A^vi^	3.0700	H15···C4^viii^	3.0000
C3···H8B^viii^	2.9700	H15···C5^viii^	2.8800
C4···H15^viii^	3.0000	H16A···C3^ii^	3.0700
C5···H3^vii^	3.0300	H16A···O1^i^	2.8600
C5···H15^viii^	2.8800	H16B···C14	2.7600
C13···H5^v^	3.1000	H16B···H14	2.3600
C14···H16B	2.7600	H16B···C15^ix^	3.0700
C14···H16C	2.7200	H16C···C14	2.7200
C15···H16B^ix^	3.0700	H16C···H14	2.2600
C15···H8B	2.9000	H16C···O2^i^	2.7800
			
C1—O2—C9	119.41 (10)	C4—C3—H3	120.00
C13—O3—C16	117.70 (12)	C3—C4—H4	120.00
O1—C1—O2	117.72 (13)	C5—C4—H4	120.00
O1—C1—C2	124.12 (13)	C4—C5—H5	120.00
O2—C1—C2	118.15 (11)	C6—C5—H5	120.00
C1—C2—C3	119.19 (12)	C5—C6—H6	120.00
C1—C2—C7	120.16 (12)	C7—C6—H6	120.00
C3—C2—C7	120.63 (12)	C7—C8—H8A	110.00
C2—C3—C4	119.71 (13)	C7—C8—H8B	110.00
C3—C4—C5	119.89 (14)	C9—C8—H8A	110.00
C4—C5—C6	120.77 (14)	C9—C8—H8B	110.00
C5—C6—C7	120.27 (13)	H8A—C8—H8B	108.00
C2—C7—C6	118.72 (12)	O2—C9—H9	106.7 (8)
C2—C7—C8	118.10 (12)	C8—C9—H9	110.0 (9)
C6—C7—C8	123.18 (12)	C10—C9—H9	110.0 (9)
C7—C8—C9	110.17 (11)	C10—C11—H11	120.00
O2—C9—C8	109.56 (10)	C12—C11—H11	120.00
O2—C9—C10	107.35 (10)	C11—C12—H12	120.00
C8—C9—C10	112.99 (11)	C13—C12—H12	120.00
C9—C10—C11	120.57 (12)	C13—C14—H14	120.00
C9—C10—C15	121.45 (12)	C15—C14—H14	120.00
C11—C10—C15	117.85 (13)	C10—C15—H15	119.00
C10—C11—C12	120.85 (14)	C14—C15—H15	119.00
C11—C12—C13	120.51 (14)	O3—C16—H16A	109.00
O3—C13—C12	116.22 (13)	O3—C16—H16B	109.00
O3—C13—C14	124.44 (13)	O3—C16—H16C	109.00
C12—C13—C14	119.34 (13)	H16A—C16—H16B	109.00
C13—C14—C15	119.47 (13)	H16A—C16—H16C	109.00
C10—C15—C14	121.98 (13)	H16B—C16—H16C	109.00
C2—C3—H3	120.00		
			
C9—O2—C1—O1	168.75 (13)	C5—C6—C7—C8	178.66 (13)
C9—O2—C1—C2	−12.42 (17)	C2—C7—C8—C9	32.06 (16)
C1—O2—C9—C8	47.27 (15)	C6—C7—C8—C9	−147.57 (13)
C1—O2—C9—C10	170.31 (11)	C7—C8—C9—O2	−55.05 (14)
C16—O3—C13—C12	175.67 (13)	C7—C8—C9—C10	−174.68 (11)
C16—O3—C13—C14	−4.8 (2)	O2—C9—C10—C11	134.82 (12)
O1—C1—C2—C3	−13.4 (2)	O2—C9—C10—C15	−49.38 (16)
O1—C1—C2—C7	164.54 (14)	C8—C9—C10—C11	−104.28 (15)
O2—C1—C2—C3	167.82 (12)	C8—C9—C10—C15	71.51 (16)
O2—C1—C2—C7	−14.21 (18)	C9—C10—C11—C12	175.05 (13)
C1—C2—C3—C4	178.77 (12)	C15—C10—C11—C12	−0.9 (2)
C7—C2—C3—C4	0.81 (19)	C9—C10—C15—C14	−175.39 (13)
C1—C2—C7—C6	−177.78 (12)	C11—C10—C15—C14	0.5 (2)
C1—C2—C7—C8	2.58 (17)	C10—C11—C12—C13	0.8 (2)
C3—C2—C7—C6	0.17 (18)	C11—C12—C13—O3	179.38 (13)
C3—C2—C7—C8	−179.48 (12)	C11—C12—C13—C14	−0.2 (2)
C2—C3—C4—C5	−1.0 (2)	O3—C13—C14—C15	−179.72 (13)
C3—C4—C5—C6	0.2 (2)	C12—C13—C14—C15	−0.2 (2)
C4—C5—C6—C7	0.8 (2)	C13—C14—C15—C10	0.0 (2)
C5—C6—C7—C2	−1.0 (2)		

Symmetry codes: (i) −*x*+2, −*y*, −*z*; (ii) *x*+1, *y*, *z*+1; (iii) *x*, *y*, *z*−1; (iv) *x*, −*y*+1/2, *z*−1/2; (v) *x*+1, *y*, *z*; (vi) *x*−1, *y*, *z*−1; (vii) *x*, −*y*+1/2, *z*+1/2; (viii) −*x*+1, −*y*, −*z*; (ix) −*x*+2, −*y*, −*z*+1; (x) *x*−1, *y*, *z*; (xi) *x*, *y*, *z*+1.

### Hydrogen-bond geometry (Å, °)

**Table:**

*D*—H···*A*	*D*—H	H···*A*	*D*···*A*	*D*—H···*A*
C8—H8B···Cg2^viii^	0.97	2.94	3.8398 (16)	154
C16—H16B···Cg3^ix^	0.96	2.89	3.7804 (17)	154

Symmetry codes: (viii) −*x*+1, −*y*, −*z*; (ix) −*x*+2, −*y*, −*z*+1.
